# Dietary Protein Source Shapes Gut Microbial Structure and Predicted Functional Potential: a Systematic Integrative Re-Analysis Using Machine Learning

**DOI:** 10.1016/j.advnut.2025.100582

**Published:** 2026-01-02

**Authors:** Samson Adedeji Adejumo, Garry Lewis, Pritha Das, Casey Kin Yun Lim, Judy Malas, Angus Nnamdi Oli, Jacob M Allen, Jarrad Hampton-Marcell

**Affiliations:** 1 Department of Biological Sciences, University of Illinois Chicago, Chicago, IL, United States; 2 Department of Health and Kinesiology, University of Illinois at Urbana-Champaign, Urbana, IL, United States; 3 Department of Pharmaceutical Microbiology and Biotechnology, Faculty of Pharmaceutical Sciences, Nnamdi Azikiwe University, Awka, Nigeria; 4 Department of Pharmaceutical Microbiology and Biotechnology, Faculty of Pharmacy, Federal University Oye-Ekiti, Oye-Ekiti, Nigeria

**Keywords:** gut microbiota, dietary protein, plant-based diet, animal-based diet, 16S rRNA sequencing, PICRUSt2, random forest, LEfSe, systematic integrative re-analysis, microbial ecology

## Abstract

Dietary proteins shape gut microbial ecology, yet the taxonomic and functional consequences of plant- compared with animal-based proteins remain poorly defined. Although digestibility and fermentation profiles differ by protein type, a systematic evaluation of how these differences influence microbial diversity, community structure, and metabolic capacity is lacking. This study represents a systematic integrative re-analysis of raw 16S rRNA sequencing datasets derived from independent controlled animal feeding studies. Following PRISMA guidelines, we analyzed 16S rRNA sequencing data from 10 murine studies (*n* = 187) comparing plant- and animal-protein diets. Alpha diversity was assessed using Shannon, Inverse Simpson, and Chao1 indices, and beta diversity with Aitchison distances. Differentially abundant taxa were identified using linear discriminant analysis, effect size, and class-weighted Random Forest (RF) models. Functional potential was inferred with phylogenetic investigation of communities by reconstruction of unobserved states, and taxon-pathway relationships were explored using correlation and network analyses. Plant-protein diets increased gut microbial diversity across all alpha diversity metrics and were associated with higher representation of saccharolytic and nitrogen-recycling genera such as *Bacteroides*, *Muribaculaceae*, and *Allobaculum*. Animal-protein diets favored proteolytic taxa, including *Clostridium sensu stricto 1* and *Colidextribacter*. Microbial community structure differed significantly between diets (analysis of similarities *R* = 0.663, *P* < 0.001). RF models achieved >88% accuracy (area under the curve = 0.995) in predicting dietary groups, and linear discriminant analysis effect size identified consistent discriminating taxa. Functional profiling showed that plant-based diets enriched pathways linked to short-chain fatty acid and aromatic amino acid metabolism, whereas animal-based diets favored sulfur- and branched-chain amino acid-associated pathways. Network analysis identified *Muribaculaceae* as a plant-associated hub and Lactobacillus as an animal-associated hub. Dietary protein source significantly influences gut microbiota composition and functional potential in mice. Plant- and animal-based proteins generate distinct metabolic signatures with implications for nitrogen cycling, sulfur metabolism, and microbial ecology. Future controlled dietary studies that harmonize protein source with other macronutrient variables are needed to isolate protein-specific effects.

**Statements of significance**

This study presents the first standardized systematic integrative re-analysis of murine protein-intervention microbiome datasets, integrating taxonomic, machine learning, and predicted functional profiling to identify robust microbial and metabolic signatures that differentiate plant- from animal-based protein diets. By harmonizing raw sequencing data across diverse experimental contexts, this work clarifies foundational ecological responses to protein source and provides mechanistic hypotheses to guide future controlled nutrition and microbiome research.

## Introduction

The gut microbiome is a complex and dynamic ecosystem that engages in bidirectional interactions with host nutrition and immunity and plays a critical role in metabolic health [1]. Although the effects of dietary carbohydrates, particularly in promoting saccharolytic fermentation and short-chain fatty acid (SCFA) production, are well established, the influence of dietary protein on gut microbial ecology is less clearly defined [2]. Dietary proteins that escape digestion in the upper gastrointestinal tract enter the colon, where they undergo fermentation by resident microbes. The outcomes of this process vary by protein source and depend on the compositional and functional capacities of the gut microbiota [3,4].

Microbial protein metabolism can proceed through saccharolytic- or proteolytic-associated pathways. Saccharolytic fermentation of amino acids can generate SCFAs that support epithelial barrier integrity and modulate host immunity [5]. In contrast, proteolytic degradation produces nitrogenous compounds such as ammonia, hydrogen sulfide (H_2_S), and phenolic metabolites. These byproducts have been associated with microbial imbalance, epithelial damage, and inflammatory responses [6,7]. Together, these distinct metabolic routes suggest that the net impact of protein fermentation is shaped by a combination of substrate availability and microbial community composition**,** and the metabolic specialization of resident taxa [8].

Importantly, not all protein sources influence the microbiota equally. Plant proteins are often accompanied by fiber and polyphenols, which foster saccharolytic fermentation and SCFA production, contributing to anti-inflammatory effects and gut barrier reinforcement [9,10]. However, plant proteins may also be less digestible and lower in certain essential amino acids (EAAs), potentially limiting their nutritional completeness [3,11,12]. In contrast, animal proteins provide a more complete amino acid profile, beneficial for muscle synthesis and maintenance, particularly in aging populations [13], but are typically higher in sulfur-containing amino acids like methionine and cysteine. These amino acids can fuel proteolytic fermentation and lead to elevated concentrations of H_2_S and ammonia, which have been associated with mucosal inflammation and impaired gut barrier function [14,15].

Despite growing interest, relatively few studies have systematically compared the microbial responses to plant-based proteins compared with animal-based proteins, particularly at both the taxonomic and functional levels. Many investigations focus on a single protein type or infer function without linking it to compositional shifts. This limits our ability to identify microbial biomarkers of protein source or to understand how these shifts contribute to gut health and disease.

Murine models remain essential for addressing these questions because they enable precise manipulation of dietary protein source while controlling for genetic background, environmental exposures, and nonprotein dietary components—conditions that are not feasible in free-living human populations [16‒18]. Such experimental consistency allows for mechanistic insights into how protein-specific perturbations structure microbial communities and their functional outputs.

To address this gap, we conducted a systematic integrative re-analysis of 16S rRNA gene amplicon sequencing data from 10 murine studies (*n* = 187), integrating statistical, machine learning, and predictive functional profiling approaches. We assessed microbial alpha and beta diversity, taxonomic composition, and classification accuracy using Random Forest (RF) and linear discriminant analysis effect size (LEfSe). Functional inference via phylogenetic investigation of communities by reconstruction of unobserved states (PICRUSt2) was used to identify enriched metabolic pathways, whereas correlation and network analyses explored taxa-function relationships. We report that plant-protein consumption is associated with greater microbial diversity and enrichment of taxa involved in SCFA production and nitrogen recycling, whereas animal protein favors the enrichment of proteolytic taxa linked to ammonia, sulfur, and phenolic metabolite production. Together, these findings offer a taxonomic and functional framework for understanding how dietary protein source modulates the gut microbiome and provide a foundation for future mechanistic studies linking diet, microbial metabolism, and host health.

## Methods

### Search strategy

This systematic integrative re-analysis followed the PRISMA guidelines [19] to ensure a transparent, systematic, and reproducible study identification and selection process. PRISMA provides a structured framework for identifying, screening, and selecting studies in a manner that minimizes bias and enhances the reliability of findings. A comprehensive literature search was conducted to identify murine studies published in different databases from inception to 31 March, 2024. Peer-reviewed, English-written articles were retrieved from *1*) Web of Science, *2*) PubMed, *3*) MEDLINE Complete, *4*) Scopus, *5*) Google Scholar, and *6*) manual searching. The Boolean search string used was: *(“protein fermentation” OR “protein digestion” OR “protein intake” OR “dietary protein” OR “protein metabolism” OR “protein consumption” OR “protein diet”) AND (“microbiome” OR “microflora” OR “microbial” OR “commensal” OR “microbiota” OR “bacteria”)” AND (“gut” OR “intestinal” OR “gastrointestinal” OR “intestine” OR “digestive tract” OR “GIT” OR “enteric” OR “ileum” OR “colon” OR “colonic” OR “duodenum” OR “jejunum” OR “fecal” OR “faecal”) AND (“mice” OR “rat” OR “rats” OR “mouse” OR “rodent” OR “rodents” OR “murine”).*

### Selection criteria

**FIGURE 1:**
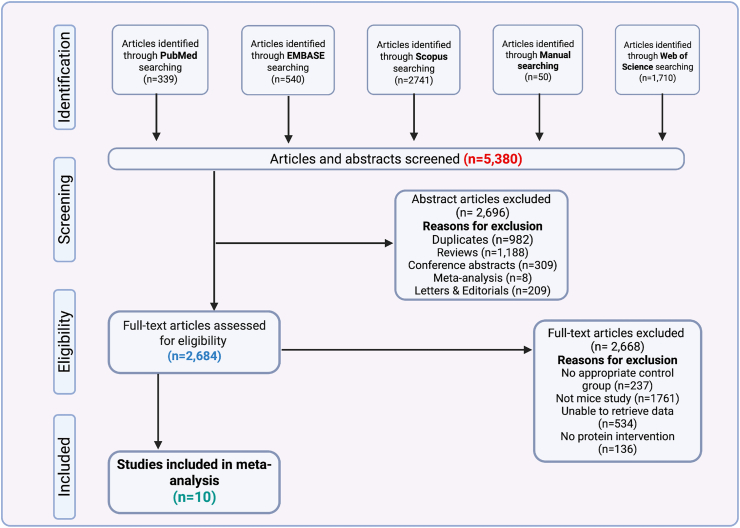
PRISMA flow diagram for study selection summarizing the systematic search and screening process for identifying eligible studies included in the analysis.

The study selection process followed PRISMA guidelines and consisted of 4 phases: *1*) identification of relevant records, *2*) screening of abstracts for eligibility, *3*) full-text eligibility assessment, and *4*) final inclusion in the systematic integrative re-analysis. The PRISMA flow diagram (Figure 1) summarizes the number of records identified, screened, excluded, and retained for analysis. Studies were included if they met the following criteria: *1*) involved murine models (mice or rats), *2*) examined dietary protein interventions, *3*) utilized 16S rRNA gene sequencing, *4*) had clearly distinguishable treatment and control groups, and *5*) provided gut microbiota composition data. Studies were excluded if they were editorials, letters, reviews, comments, or other meta-analyses, lacked a protein intervention, involved nonmurine or in vitro studies, or had unpublished data or unclear dietary group differentiation. Search results were exported to Mendeley for initial deduplication, followed by a second deduplication in Zotero. The remaining records were manually screened by title and abstract to exclude studies irrelevant to the research objectives.

### Bioinformatics and data processing

Raw sequencing data (FASTQ files) were retrieved from the National Center for Biotechnology Information and European Nucleotide Archive repositories using sequence read archive accession numbers reported in the included studies. Amplicon sequence processing was conducted using quantitative insights into microbial ecology 2-amplicon-2024.5 [20,21]. Reads were demultiplexed and quality-filtered using the q2-demux plugin, followed by denoising with divisive amplicon denoising algorithm 2 (v1.28.0) to generate high-resolution amplicon sequence variants (ASVs) [22]. Taxonomic assignment was performed against the SILVA ribosomal RNA (rRNA) gene reference database (release 138) using a Naïve Bayes classifier implemented via the q2-feature-classifier plugin [23]. ASVs with confidence scores <70% were discarded to improve taxonomic reliability.

Processed data, including feature tables, phylogenetic trees, and taxonomic classifications, were imported into RStudio (v4.4.1; Posit Software, Boston, MA, USA) for downstream analyses. Microbiome data were integrated using the phyloseq package (v1.48.0; Bioconductor project, maintained by Paul J. McMurdie and Susan Holmes), and statistical analyses were conducted using ggplot2 (v3.5.1), patchwork (v1.3.0), ggpubr (v0.6.0), pheatmap (v1.0.12), and dplyr (v1.1.4) [24,25].

Functional metagenomic prediction was performed using PICRUSt2 (v2.5.2), which infers Kyoto Encyclopedia of Genes and Genomes (KEGG) Orthologs (KOs) and MetaCyc Metabolic Pathway Database pathways from 16S rRNA gene data via evolutionary modeling. The predicted KO and pathway tables were normalized to relative abundance and exported for downstream differential abundance testing and network analysis.

Among the included studies, 9 targeted the V3-V4 region of the 16S rRNA gene using either the 338F/806R or 515F/806R primer pairs. Both forward primers amplify the V3 region, but 338F (5′-ACTCCTACGGGAGGCAGCAG-3′) is known for its broad bacterial coverage in murine gut microbiota, whereas 515F (5′-GTGCCAGCMGCCGCGG-3′) is commonly used in high-throughput studies due to its compatibility with barcoding and multiplex sequencing [26]. The reverse primer 806R (5′-GGACTACHVGGGTWTCTAAT-3′) was used consistently across all V3-V4 studies. One study amplified the V1-V2 region using 27F (5′-AGAGTTTGATCMTGGCTCAG-3′) and 338R (5′-TGCTGCCTCCCGTAGGAGT-3′), which target a more upstream and variable portion of the gene. Primer selection can influence sequencing depth, taxonomic bias, and classification accuracy [26,27], so all primer sequences and target regions are summarized in Supplementary Table 1.

### Statistical analysis

Given the diversity of experimental designs across included studies, all statistical analyses were conducted under a framework that explicitly models inter-study heterogeneity rather than assuming equivalence among datasets. Mixed-effects approaches incorporating study-level variables, including animal age, anatomical sampling site, country of origin, and dietary protein concentration, were used where appropriate to account for contextual variability. This strategy enables identification of protein-source–associated microbial patterns that recur across heterogeneous experimental settings while minimizing study-specific artifacts.

#### Data transformation

Centered log-ratio (CLR) transformation was applied to genus-level relative abundance tables before all compositional analyses. CLR normalization addresses the inherent compositional constraints of microbiome data by expressing abundances as log-ratios, thereby reducing spurious correlations and enabling valid Euclidean distance–based comparisons [28,29]. All CLR-based outputs in this study, including Aitchison distances and enrichment estimates, were generated using the microbiome and phyloseq packages in R.

#### Handling of missing data

To ensure consistency across datasets, microbiome features with 0 counts were retained as biological zeros rather than treated as missing values. Samples lacking essential metadata (e.g., protein concentrations, animal age, country of origin, or sample isolation site) were excluded only from analyses that required those variables as fixed or random effects but were retained in descriptive summaries when appropriate. For functional pathway data, features with >20% missing values across studies were removed prior to analysis, and remaining missing entries were imputed as zeros, consistent with conventions in compositional microbiome analysis [28]. These steps minimized bias arising from incomplete metadata while preserving the integrity of taxonomic and functional profiles across studies.

#### Alpha and beta diversity

Alpha diversity indices (Shannon, Inverse Simpson, and Chao1) were used to assess microbial richness and diversity [[30], [31], [32]]. Comparisons between plant- and animal-protein diets were performed using the Wilcoxon Rank-Sum test. Beta diversity was assessed using Aitchison distance (Euclidean distance on CLR-transformed data) and visualized via principal component analysis (PCA). Analysis of similarities (ANOSIM) tested whether microbial composition varied more between dietary groups than within groups [33]. Permutational analysis of variance (PERMANOVA) was conducted to quantify the variance in microbial composition explained by dietary protein sources [34].

#### Generalized linear mixed model

To account for inter-study heterogeneity and sampling variability, a generalized linear mixed model (GLMM) was implemented using the lme4 (v1.1-34) package in R. Shannon diversity served as the continuous outcome variable, enabling estimation of the independent association between dietary protein source and alpha diversity. Dietary protein source and protein concentration were modeled as fixed effects, whereas random intercepts were specified for country of origin, sample isolation site (fecal, colonic, ileal, and small intestine), and animal age (in weeks) to control for study-level and anatomical variation. Different model configurations were evaluated, and final model selection was guided by goodness-of-fit statistics, including Akaike information criterion and Bayesian information criterion. This modeling approach enabled robust estimation of microbial diversity metrics while accounting for clustering and potential confounding across datasets [35,36].

#### Machine learning classification and microbial feature selection

To classify dietary protein sources and identify microbial biomarkers, a supervised RF model was implemented using the randomForest package in R [37]. Three RF approaches were compared: *1*) unadjusted RF, trained on the original imbalanced dataset; *2*) class-weighted RF, which accounted for class imbalance by assigning inverse frequency weights to underrepresented groups; and *3*) synthetic sampling RF, using the ROSE (Random OverSampling Examples) technique to balance the dataset.

Microbial relative abundance data at the genus level were used as input features. The dataset was randomly partitioned into training (70%) and testing (30%) sets. Model performance was evaluated using the out-of-bag error rate, confusion matrices, and area under the receiver operating characteristic (ROC) curve. Feature importance was determined using the mean decrease in accuracy, which ranked genera based on their contribution to classification performance.

To complement the RF analysis, LEfSe was performed using the microbiomeMarker package (v1.10.0) in R to independently identify microbial taxa significantly enriched in plant- compared with animal-protein diets. LEfSe was applied to genus-level relative abundance data, using a linear discriminant analysis score threshold >3.0 and adjusted *P* value <0.05. A taxonomic cladogram was generated to visualize differentially enriched microbial features between dietary groups [38].

#### ROC analysis

ROC analysis was used to assess the predictive performance of the 3 RF models. ROC curves were generated using the pROC package (v1.18.0) in R. Each model was trained to classify diets based on microbial genus-level profiles, distinguishing between plant and animal-protein sources. Predictions from the test dataset were used to generate ROC curves, and the AUC was computed to quantify model performance. Higher AUC values indicated stronger sensitivity-specificity trade-offs and improved classification capability. Overall classification accuracy was also reported to compare performance across sampling strategies.

ROC analysis is commonly used in microbiome studies to assess and classify cation models, particularly in dietary and clinical interventions [39]. Evaluating multiple sampling strategies—including the original data distribution, class weighting, and synthetic resampling—provides a more robust assessment of model reliability and reduces the risk of overfitting [40].

#### Functional profiling and pathway analysis

Functional metagenomic predictions were generated using PICRUSt2 [41], which infers gene family abundances from 16S rRNA data by leveraging ancestral-state reconstruction of known reference genomes. KOs were predicted and mapped to MetaCyc and KEGG pathway hierarchies to enable downstream pathway-level analysis.

Differential pathway abundance between plant- and animal-protein diets was analyzed using MaAsLin2 (Multivariable Association with Linear Models 2) [42], a multivariable linear modeling approach that allows covariate adjustment and correction for multiple testing. Relative abundances were log-transformed, and protein source was included as a fixed effect. Pathways with false discovery rate–adjusted *P* values < 0.05 were considered statistically significant.

To visualize functional differences, heatmaps were generated using the pheatmap R package, focusing on the top-ranked differentially abundant pathways. Additional analysis focused on metabolic pathways related to sulfur metabolism, SCFA production, and nitrogen cycling. Taxa and pathway relationships were examined using Spearman correlation, and network topology metrics such as node centrality and clustering coefficients were used to characterize ecological interactions.

#### Network analysis

To investigate ecological interactions between microbial taxa and predicted functional pathways, correlation-based co-occurrence networks were constructed. Genus-level relative abundances and KO predicted by PICRUSt2 were used as input. Pairwise Pearson correlation coefficients were computed, and significant associations were retained based on a *P* value threshold of < 0.05 and an absolute correlation coefficient (|r|) ≥0.4, consistent with prior network-based microbiome studies [43,44].

Networks were visualized using the igraph and ggraph R packages. In each network, nodes represented microbial genera or functional pathways, and edges indicated significant positive or negative correlations. Edge thickness corresponded to the strength of the correlation.

To further characterize the structure of these networks, topological features were assessed using centrality measures, including *degree*, *betweenness*, and *eigenvector centrality*. Degree centrality reflects the number of direct connections a node has, indicating its immediate influence or connectivity within the network. Betweenness centrality captures how often a node lies on the shortest paths between other nodes, thus highlighting its role as a potential bridge or bottleneck in network flow. Eigenvector centrality assigns importance based not only on the number of connections but also on the influence of connected neighbors, identifying nodes with broader systemic influence. These measures were used to identify potential keystone taxa and central metabolic pathways that may play critical roles in shaping the microbiome’s structure and function [45,46].

This integrative approach allowed for the simultaneous analysis of microbial community structure and functional potential, revealing clusters of co-associated taxa and pathways shaped by dietary protein source.

### Cross-species comparison between human and murine microbiota

This cross-species analysis was designed to provide contextual insight into the prevalence and distribution of protein-responsive microbial genera in human populations, rather than to test cross-species concordance of dietary effects. Accordingly, the human microbiome analysis should not be interpreted as validation of murine findings, and no direct equivalence between murine and human dietary responses is assumed.

To evaluate whether microbial patterns identified in mice reflect broader trends in humans, we performed a cross-species comparison using stool microbiome data from the American Gut Project (AGP) [47, 1[47], 1 of the largest publicly available human 16S rRNA datasets. Approximately 20,000 AGP samples were reprocessed using the same analytical workflow applied to the murine data. Human FASTQ files were denoised with divisive amplicon denoising algorithm 2 [22], taxonomic assignment was performed using the SILVA 138 reference database [27], and CLR transformation was applied to normalize compositional abundances [28,29]. Matching these preprocessing steps minimized technical variability between species.

Shared genera were identified from the CLR-transformed human and murine datasets and used to compute 3 complementary indicators of cross-species similarity: *1*) mean relative abundance and prevalence of each shared genus in the AGP population, *2*) correspondence in CLR-transformed abundance between species using Spearman correlation, and *3*) genus-level effect-size differences expressed as ΔCLR (human − mouse). Murine CLR values were derived from the high-protein diet cohort to provide a diet-aligned reference for comparison. All analyses were performed in R (v4.4.1) using phyloseq [24], microbiome [48], dplyr [25], and ggplot2 [49], with identical filtering and normalization steps applied across datasets to ensure methodological consistency.

## Results

### Characteristics of selected studies

The systematic search identified a total of 5380 articles from PubMed (*n* = 339), EMBASE (*n* = 540), Scopus (*n* = 2,741), Web of Science (*n* = 1,710), and manual searches (*n* = 50). After deduplication and preliminary screening, 2696 abstracts were excluded due to duplicates (*n* = 982), review articles (*n* = 1188), conference abstracts (*n* = 309), meta-analyses (*n* = 8), and letters or editorials (*n* = 209).

Subsequently, 2684 full-text articles were assessed for eligibility. Of these, 2668 were excluded for the following reasons: lack of an appropriate control group (*n* = 237), use of non-murine models (*n* = 1761), inability to retrieve sequencing data (*n* = 534), or absence of a defined protein intervention (*n* = 136).

Ultimately, 10 studies met all inclusion criteria and were included in the systematic integrative re-analysis [[50], [51], [52], [53], [54], [55], [56], [57], [58], [59]] (Table 1). These studies provided accessible 16S rRNA sequencing data, clearly defined animal- or plant-based protein interventions, and complete sample metadata. To improve transparency in dietary comparisons, we summarized the macronutrient composition (percentage kilocalories from protein, fat, and carbohydrates, including fiber) for all included rodent diets in Supplementary Table 2. Although 9 distinct dietary protein sources were represented—branched-chain amino acids (BCAAs), beef, casein, chicken, EAAs, low-protein diets, pork, sausage, and soy protein—they were categorized into 2 main groups for analysis: animal-based proteins (beef, casein, chicken, pork, sausage, and BCAAs/EAAs when derived from animal sources) and plant-based proteins (soy and low-protein diets, as applicable). This binary classification enabled a focused comparison of microbial responses to protein origin across studies. A detailed breakdown of sample distribution by protein type and protein source is shown in Supplementary Figure 1. The study selection process is summarized in Figure 1.

**Table 1:** Characteristics of studies included in the systematic integrative re-analysis

Country, year, first author	Title	Bioproject accession	Protein source	Sample no.	Age of mice (wk)
Australia, 2022, Jian Tan	Dietary protein increases T-cell-independent sIgA production through changes in gut microbiota-derived extracellular vesicles	PRJEB39583	Casein	8	6
Japan, 2020, Hiroaki Masuoka	The influences of low-protein diet on the intestinal microbiota of mice	PRJDB8898	Casein	30	9
China, 2022, Yantao Yin	Dietary oxidized beef protein alters gut microbiota and induces colonic inflammatory damage in C57BL/6 mice	PRJNA872483	Oxidized beef	24	5
China, 2022, Yifan Zhu	Reno-protective effect of low-protein diet supplemented with a-ketoacid through gut microbiota and fecal metabolism in 5/6 nephrectomized mice	PRJNA810785	Low-protein diet	10	8
China, 2020, Yunting Xie	Processing method altered mouse intestinal morphology and microbial composition by affecting the digestion of meat proteins	PRJNA607338	Casein, pork, sausage, soy protein	30	4
Canada, 2021, B.S.-Y. Choi	Feeding diversified protein sources exacerbates hepatic insulin resistance via increased gut microbial branched-chain fatty acids and mTORC1 signaling in obese mice	PRJEB37442	Casein	12	10
China, 2020, Muhammad Umair Ijaz	Meat protein in a high-fat diet induces adipogenesis and dyslipidemia by altering gut microbiota and endocannabinoid dysregulation in the adipose tissue of mice	PRJNA548036	Beef, casein, chicken, pork	27	7
China, 2022, Wuling Zhong	High-protein diet prevents fat mass increase after dieting by counteracting Lactobacillus-enhanced lipid absorption	PRJNA868153	Casein	14	8
China, 2022, Ranran Zhang	Oral administration of branched-chain amino acids ameliorates high-fat diet-induced metabolic-associated fatty liver disease via gut microbiota-associated mechanisms	PRJNA852193	Branched-chain amino acids	12	6
Italy, 2020, Chiara Ruocco	Manipulation of dietary amino acids prevents and reverses obesity in mice through multiple mechanisms that modulate energy homeostasis	PRJEB25686	Essential amino acids, casein	20	8

Abbreviations: mTORC1, mechanistic target of rapamycin complex 1; sIgA, secretory immunogloblin A.

### Composition of core gut bacteria in protein-fed mice

A total of 9,575,175 sequencing reads were generated from 187 protein-fed mouse samples, yielding 16,630 ASVs, with an average of 51,204 sequence reads per sample. After denoising and chimera removal, 10,123 ASVs remained. Across all samples, Firmicutes (58.48%) and Bacteroidota (21.88%) were the dominant phyla, followed by Proteobacteria (7.08%), Actinobacteriota (4.13%), Acidobacteriota (1.34%), and Verrucomicrobiota (1.28%). The Firmicutes to Bacteroidota (F: B) ratio, a traditional indicator of metabolic health, was significantly higher in animal-protein–fed mice (63.3:19.9) compared with plant-protein–fed mice (47.3:23.5) (Supplementary Figure 2, Supplementary Table 3).

**FIGURE 2:**
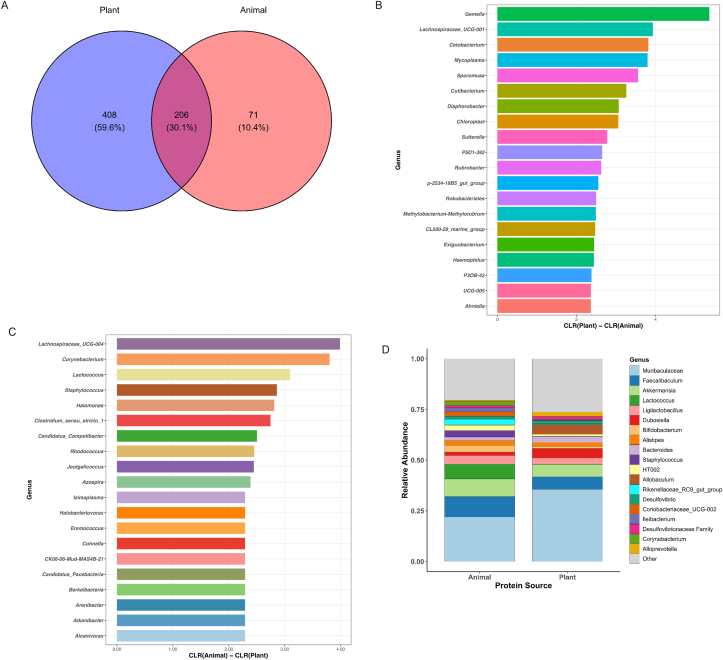
Microbial composition and taxonomic uniqueness across dietary protein sources. (A) Venn diagram showing the number and percentage of microbial genera unique to plant-fed and animal-fed mice, as well as genera shared between both groups. (B) Top genera enriched in plant-protein diets, displayed as CLR(Plant) − CLR(Animal) differences. Positive values indicate higher normalized abundance under plant-protein intake. (C) Top genera enriched in animal-protein diets, displayed as CLR(Animal) − CLR(Plant) differences. Positive values indicate higher normalized abundance under animal-protein intake. (D) Stacked bar plot comparing the relative abundance of dominant genera across dietary groups, highlighting taxa that differ consistently between plant- and animal-protein diets. CLR, centered log-ratio.

Among the 685 identified genera, plant-protein diets supported a significantly higher number of unique microbial taxa (408 genera, 59.6%) compared with animal-protein diets (71 genera, 10.4%) (Figure 2A), indicating greater microbial diversity. Despite this difference, both dietary groups shared a core set of dominant taxa, with *Muribaculaceae*, *Faecalibaculum*, and *Akkermansia* highly abundant across all samples, although their relative abundance varied by diet. The relative abundance of dominant genera stratified by animal- and plant-based protein diets is presented in Table 2.

**TABLE 2:** Core microbiota based on protein source at genus level

Genera	Animal protein (%)	Genera	Plant protein (%)
*Muribaculaceae*	21.93	*Muribaculaceae*	35.64
*Faecalibaculum*	10.01	*Faecalibaculum*	6.17
*Akkermansia*	8.70	*Akkermansia*	6.02
*Lactococcus*	7.34	*Allobaculum*	4.79
*Ligilactobacillus*	4.24	*Dubosiella*	4.79
*Staphylococcus*	3.25	*Bacteroides*	3.02
*Bifidobacterium*	3.21	*Ligilactobacillus*	2.99
*Rikenellaceae_RC9_gut_group*	2.82	*Alloprevotella*	2.15
*HT002*	2.49	*Alistipes*	1.84
*Alistipes*	2.41	*Desulfovibrio*	1.57
Others	20.44	Others	26.27

To quantify how unique taxa were distributed across diets, we evaluated CLR enrichment among genera detected exclusively within each group. Plant-unique taxa exhibited uniformly positive CLR differences (CLR^*Plant*^ − CLR^*Animal*^), indicating consistent enrichment under plant-protein feeding (Figure 2B). The most strongly represented genera included *Gemella, Lachnospiraceae UCG-001, Cetobacterium, Mycoplasma,* and *Sporomusa,* along with several low-abundance fermentative taxa that contributed to the broader ecological repertoire of plant-fed microbiota. Conversely, genera unique to animal-protein diets displayed positive CLR differences in the opposite direction (CLR^*Animal*^ − CLR^*Plant*^), reflecting preferential representation under animal-derived proteins (Figure 2C). Key contributors included *Lachnospiraceae UCG-004, Corynebacterium, Lactococcus, Staphylococcus, Halomonas,* and *Clostridium sensu stricto 1,* many of which are associated with proteolytic metabolism.

Beyond unique genera, relative abundance comparisons of shared taxa revealed clear dietary signatures (Figure 2D). Plant-protein diets were characterized by higher levels of saccharolytic fermenters, including *Muribaculaceae* (35.64% compared with 21.93% in animal-fed mice), *Dubosiella* (4.79% compared with 2.11%), and *Allobaculum* (4.79% compared with 1.42%), reflecting enhanced utilization of plant-derived substrates. In contrast, animal-protein diets showed increased representation of proteolytic taxa such as *Lactococcus* (7.34% compared with 0.82% in plant-fed mice), *Ligilactobacillus* (4.24% compared with 1.03%), and *Staphylococcus* (3.25% compared with 0.56%), genera commonly associated with protein fermentation and nitrogen-rich metabolic byproducts. These differences highlight distinct compositional patterns associated with each protein source, with plant-derived proteins showing higher abundance of saccharolytic taxa and animal-derived proteins showing greater representation of proteolytic genera.

### Dietary protein sources modulate microbial diversity

**FIGURE 3:**
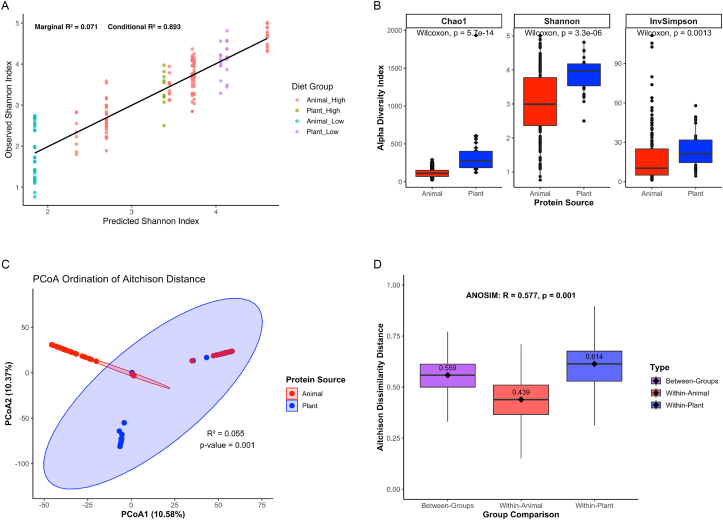
Microbial diversity and community structure in protein-based diets. (A) Linear mixed model comparing predicted vs. observed Shannon diversity, with marginal and conditional *R*^*2*^ values indicating variance explained by fixed effects alone (0.071) vs. fixed plus random effects (0.893), respectively. (B) Boxplots showing significantly higher microbial richness and evenness in plant-based protein diets. (C) Principal coordinates analysis (PCoA) based on Aitchison distance reveals clear separation of microbial communities by protein source. Ellipses represent 95% confidence intervals. (D) ANOSIM analysis confirms significant differences in microbial community structure between diet groups (R = 0.663, P < 0.001). ANOSIM, analysis of similarities; InvSimpson, Inverse Simpson.

To assess how dietary protein source, protein concentration, and other contextual factors affect microbial diversity, we employed a GLMM framework as described in Section Handling of missing data. The best-fitting model included dietary protein source and protein concentration as fixed effects, with random intercepts specified for country of origin, sample isolation site, and animal age (in weeks). This model achieved the lowest Akaike information criterion (252.88) and Bayesian information criterion (278.20), and the highest log-likelihood (−118.44) (Supplementary Table 4). Model selection was further validated by likelihood ratio tests and *R*^*2*^ metrics, revealing a marginal *R*^*2*^ of 0.071 and a conditional *R*^*2*^ of 0.893, indicating substantial contribution from both fixed and random effects (Figure 3A). Protein source had the strongest positive association with Shannon diversity (β = 1.25), followed by protein concentrations (β = 0.40). Sequencing depth did not significantly impact diversity estimates (ρ = 0.107, *P* = 0.145), suggesting biological rather than technical drivers of variation (Supplementary Figure 3A).

Consistent with these model outputs, microbial alpha diversity was significantly higher in plant-protein diets across all 3 diversity indices: Chao1 (*P* = 5.7 × 10^-14^), Shannon (*P* = 3.3 × 10^-6^), and Inverse Simpson (*P* = 0.0013) (Figure 3B). A notable interaction between protein source and concentration was detected (Supplementary Figure 3B), with Shannon diversity highest in high plant-protein diets (*P* = 7.39 × 10^-15^), whereas differences were not significant at low-protein concentrations (*P* = 0.722). These results suggest that both the source and concentration of dietary protein contribute to microbial richness and evenness.

To further examine shifts in overall microbial community composition, we analyzed beta diversity using Aitchison distance–based PCA. The ordination revealed distinct clustering by protein source, with the first 2 components explaining 10.58% and 10.37% of the total variance, respectively (Figure 3C). These compositional differences were confirmed by PERMANOVA (*R*^*2*^ = 0.055, *P* = 0.001) and ANOSIM (*R* = 0.663, *P* = 0.001), underscoring significant divergence in microbial communities between plant- and animal-protein diets (Figure 3D).

Together, these findings demonstrate that dietary protein source exerts a dominant influence on both within-sample (alpha) and between-sample (beta) microbial diversity, independent of sequencing depth or study-specific factors. High plant-protein intake supports a more diverse and compositionally distinct gut microbiota, implicating protein source as key in modulating the gut microbial ecosystem.

### Machine learning identifies microbial predictors of protein source

**FIGURE 4:**
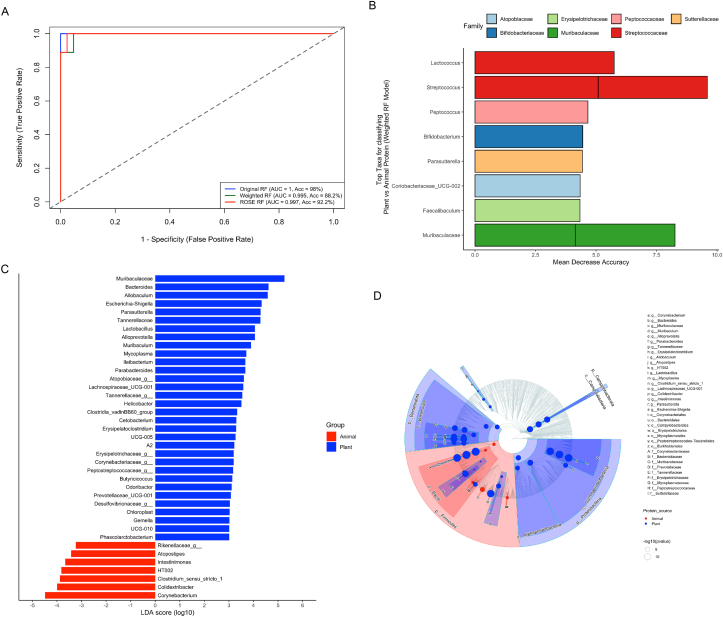
Machine Learning performance and key microbial predictors of dietary protein source. (A) Receiver operating characteristic (ROC) curves showing percentage accuracy and AUC comparing 3 Random Forest (RF) models: unadjusted (original), class-weighted (weighted), and balanced via ROSE sampling. The weighted RF model, although slightly lower in accuracy, avoids overfitting and offers more realistic performance, with an AUC of 0.995 and accuracy of 88.2%. (B) Top 10 microbial genera contributing most to the weighted RF model’s classification of animal vs. plant-protein diets, based on mean decrease in accuracy. (C). LDA using LEfSe highlights taxa enriched in each diet (LDA score ≥3.0, *P* < 0.05) (D). Cladogram shows microbial taxa enriched in plant-based (blue) and animal-based (red) diets, arranged hierarchically from phylum to genus with node sizes reflecting statistical significance [‒log10 (*P* value)]. AUC, area under the curve; LDA, linear discriminant analysis; LEfSe, linear discriminant analysis effect size; ROSE, Random OverSampling Examples.

Supervised machine learning confirmed that gut microbial composition is highly predictive of dietary protein source. Among the 3 RF models evaluated, the unadjusted RF yielded the highest classification performance (AUC = 1.00, accuracy = 98%), followed by the ROSE-sampled RF (AUC = 0.997, accuracy = 92.2%) and the class-weighted RF (AUC = 0.995, accuracy = 88.2%) (Figure 4A).

Despite its slightly lower accuracy, the class-weighted model was selected for downstream feature selection to correct for class imbalance between plant- and animal-protein groups. Feature importance analysis based on mean decrease in accuracy identified key taxa contributing to classification, including *Lactococcus* (5.34%), *Streptococcus* (5.09%, 4.34%), *Peptococcus* (4.71%), *Bifidobacterium* (4.56%), *Parasutterella* (4.47%), *Faecalibaculum* (4.43%), *Coriobacteriaceae UCG-002* (4.44%), and *Muribaculaceae* (4.10%, 4.06%) (Figure 4B; Supplementary Table 5).

Model robustness was supported by cross-validation and comparison with a randomized control model, which achieved only 38.2% accuracy (Supplementary Figure 4A). The error-per-tree diagnostic further confirmed model stability across 1,000 trees (Supplementary Figure 4B). Confusion matrix analysis showed high specificity for plant-protein predictions, with modest misclassification among animal-protein samples (Supplementary Figure 4C).

LEfSe analysis further validated these findings, identifying 33 genera enriched in plant-protein diets and 7 in animal-protein diets. Notably, plant-associated genera included *Muribaculaceae, Bacteroides, Parabacteroides,* and *Allobaculum*, whereas animal-protein diets were characterized by genera such as *Clostridium sensu stricto* 1*, Colidextribacter,* and *Rikenellaceae.* A LEfSe-derived cladogram illustrated the phylogenetic structure of these associations, with plant-protein diets favoring Bacteroidota and Proteobacteria, and animal-protein diets skewed toward Firmicutes dominance (Figure 4C and D; Supplementary Table 6). These results demonstrate the use of machine learning for uncovering diet-responsive microbial biomarkers and highlight the biological relevance of microbial shifts linked to dietary protein type.

### Functional pathways enriched by protein type

**FIGURE 5:**
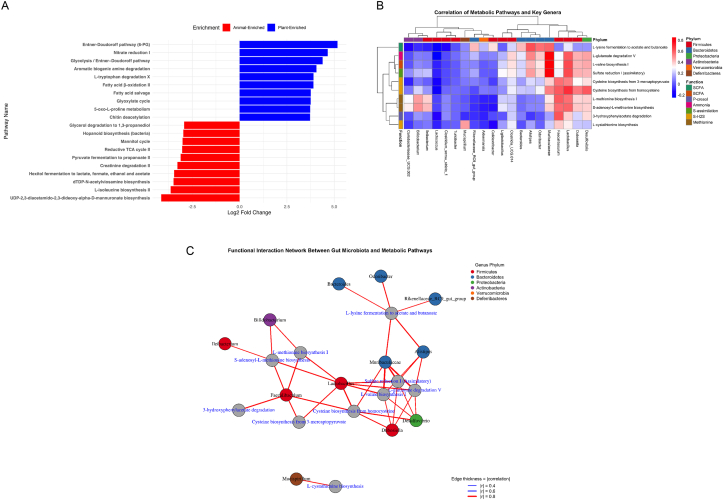
Functional profiling of microbial communities by protein source using PICRUSt2 predictions. (A) Top 10 KEGG pathways significantly enriched in mice fed animal- or plant-based protein diets (|log_2_ fold change| >2, FDR <0.05). Red bars indicate enrichment in the animal group; blue bars indicate enrichment in the plant group. (B) Heatmap showing Pearson correlation between microbial genera and selected predicted metabolic pathways involved in short-chain fatty acid (SCFA), branched-chain fatty acid (BCFA), phenol/p-cresol, ammonia, and sulfur metabolism. Functions are color-coded by metabolic category, and genera are annotated by phylum. (C) Network diagram of strong correlations (|r| ≥0.6) between microbial genera and selected predicted metabolic pathways. Node size reflects the number of connections (degree centrality), and edge thickness corresponds to the strength of correlation. FDR, false discovery rate; KEGG, Kyoto encyclopedia of genes and genomes; PICRUSt2, phylogenetic investigation of communities by reconstruction of unobserved states.

Functional profiling using PICRUSt2 revealed distinct metabolic signatures between plant- and animal-protein diets. Differential pathway enrichment analysis identified 10 plant-enriched and 10 animal-enriched MetaCyc pathways with significant log_2_ fold changes (Figure 5A). Plant-based diets were associated with pathways related to carbohydrate metabolism and amino acid degradation, including the Entner-Doudoroff pathway, nitrate reduction, aromatic biogenic amine degradation, and L-tryptophan degradation. Conversely, animal-protein diets were enriched in fermentation and biosynthesis pathways such as creatinine degradation, propanoate fermentation, and L-isoleucine biosynthesis. These findings highlight distinct metabolic capacities of the microbiota shaped by dietary protein source.

### Correlation and network analysis reveal functional interactions

To investigate how enriched pathways relate to specific microbial taxa, a correlation analysis was performed between predicted KO functions and the top differential genera. The resulting heatmap (Figure 5B) highlighted strong associations between specific microbial genera and functional pathways involved in SCFA, branched-chain fatty acid (BCFA), sulfur, and amino acid metabolism. *Lactobacillus* correlated with cysteine biosynthesis (*r* = 0.693, *P* = 6.5 × 10^-28^), sulfate reduction (*r* = 0.647, *P* = 7.1 × 10^-23^), and methionine biosynthesis (*r* = 0.566, *P* = 2.4 × 10^-17^). *Faecalibaculum* showed positive correlations with methionine biosynthesis (*r* = 0.606, *P* = 1.4 × 10^-20^) and cysteine biosynthesis (*r* = 0.602, *P* = 2.7 × 10^-20^). *Dubosiella* was linked to glutamate degradation (*r* = 0.440, *P* = 2.5 × 10^-10^) and cysteine biosynthesis (r = 0.537, *P* = 1.8 × 10^-15^). *Bifidobacterium* was moderately correlated with methionine pathways, including S-adenosylmethionine biosynthesis (*r* = 0.503, *P* = 3.8 × 10^-13^). In contrast, *Alistipes* and *Muribaculaceae* were associated with SCFA-related pathways, including lysine fermentation (*r* = 0.619, *P* = 8.5 × 10^-22^ and *r* = 0.582, *P* = 5.6 × 10^-19^, respectively), glutamate degradation (*r* = 0.509, *P* = 2.3 × 10^-14^ and *r* = 0.855, *P* = 2.1 × 10^-54^), and valine biosynthesis (*r* = 0.422, *P* = 4.3 × 10^-10^ and *r* = 0.858, *P* = 7.6 × 10^-55^). These patterns suggest that distinct microbial taxa specialize in different metabolic niches depending on dietary protein source.

Network analysis based on pairwise Pearson correlations (|r| ≥0.4, *P* < 0.05) visualized the co-occurrence structure of microbial taxa and metabolic functions (Figure 5C). Nodes represented taxa or pathways, and edge thickness indicated correlation strength. Highly connected nodes such as *Faecalibaculum* (eigenvector centrality = 0.97) and *Lactobacillus* (0.97) exhibited strong topological influence within sulfur and methionine metabolic subnetworks, suggesting they may serve as functional keystone taxa in modulating amino acid–related metabolic functions.

Similarly, *Muribaculaceae* demonstrated the highest eigenvector centrality (1.00) (Supplementary Figure 5), underscoring its potential integrative role across diverse metabolic modules, particularly those involved in glutamate degradation and SCFA production. These network metrics reinforce the centrality of select taxa in shaping key nutrient pathways influenced by dietary protein source.

### Cross-species comparison of shared gut bacterial genera

**FIGURE 6:**
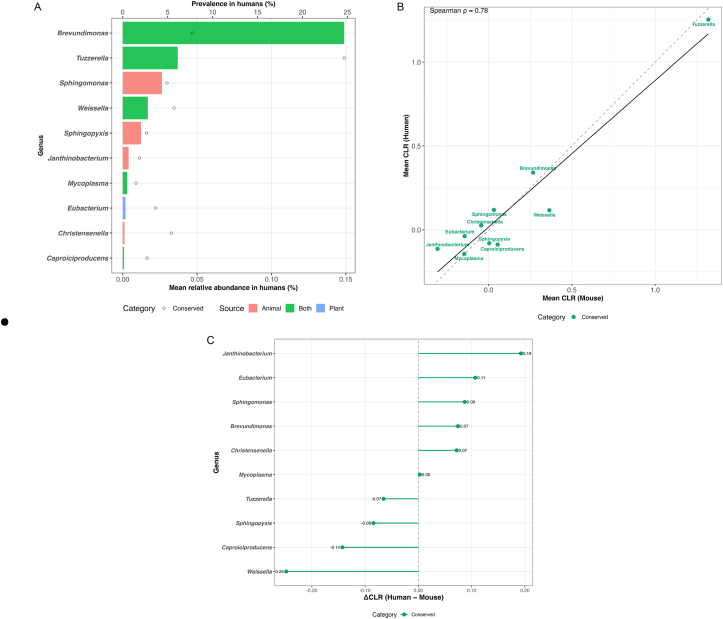
Cross-species compositional similarity of gut bacterial genera shared between humans and mice. (A) Mean relative abundance and prevalence of genera detected in both humans and mice; bars represent human relative abundance grouped by source type (animal-, plant-associated, or mixed), and points indicate human population prevalence. (B) Cross-species mean CLR per genus; points represent shared genera in humans and mice. The dashed diagonal denotes perfect correspondence (y = x), and the solid line shows the regression trend (Spearman ρ = 0.78). (C) CLR effect-size differences (ΔCLR = human − mouse). The lollipop plot displays direction and magnitude of cross-species shifts, where values near 0 indicate similar ecological representation in both hosts. CLR, centered log-ratio; H_2_S, hydrogen sulfide.

A subset of genera detected in the murine datasets also appeared in the AGP cohort, demonstrating that several protein-responsive taxa are not restricted to mice. Among shared genera, *Brevundimonas* (24.6% prevalence) and *Tuzzerella* (7.8%) were the most frequently observed in humans, with additional contributions from *Weissella, Sphingomonas, Sphingopyxis, Christensenella,* and *Eubacterium* (Figure 6A).

CLR-transformed abundance profiles showed a positive cross-species correspondence (Spearman ρ = 0.78), indicating that genera with higher CLR values in mice tended to occupy similar positions along the abundance gradient in humans (Figure 6B).

Genus-level effect-size differences (ΔCLR = human − mouse) were modest and generally centered near 0 (Figure 6C). *Janthinobacterium* and *Eubacterium* showed slightly elevated CLR values in humans, whereas *Weissella, Caproiciproducens,* and *Sphingopyxis* displayed higher CLR values in mice. Despite these differences, most genera fell within overlapping CLR ranges, reflecting broad consistency in their comparative abundance across hosts.

Together, these results indicate that several taxa enriched under protein-modulated conditions in mice are also represented in human stool samples, with broadly similar abundance patterns across species.

## Discussion

This study integrates harmonized 16S rRNA datasets from multiple murine dietary protein-intervention studies to examine how dietary protein source is associated with gut microbial composition, diversity, and inferred metabolic potential across heterogeneous experimental contexts. Rather than pooling reported outcomes or estimating a single summary effect size, the analysis is based on systematic reprocessing of raw sequencing data using a unified bioinformatic and statistical framework. This integrative approach enables identification of microbial taxa and predicted metabolic pathways that recur across independent studies, highlighting reproducible ecological patterns associated with protein source. Across datasets, plant- and animal-derived proteins were consistently associated with distinct microbial assemblages and functional tendencies, reflecting differences in nutrient availability, fermentation substrates, and nitrogen handling within the gut ecosystem. Together, these findings advance understanding of how dietary nitrogen and carbon inputs structure microbial communities and organize their metabolic potential, providing an ecological framework for interpreting diet-microbiome interactions relevant to host physiology.

### Overview of diet-associated taxonomic patterns

Dietary protein source was associated with clearly differentiated taxonomic profiles in the murine gut microbiota. Plant-derived protein diets supported a broader range of microbial taxa, including genera characterized by saccharolytic metabolism and lower nitrogen demand, consistent with the expanded ecological niches afforded by plant-based substrates. In contrast, animal-derived protein diets favored taxa adapted to proteolytic metabolism and nitrogen-rich environments. These patterns align with established ecological principles describing how substrate composition and nitrogen availability drive niche partitioning and competitive dynamics within microbial communities [6,60].

CLR-based enrichment analyses further reinforced this divergence, with plant-associated genera exhibiting higher relative abundance under plant-protein feeding and animal-associated genera showing reciprocal enrichment under animal-protein conditions. The consistency of these trends across studies suggests that dietary protein source establishes distinct resource landscapes within the gut that systematically select for microbial lineages with corresponding metabolic strategies. These taxonomic patterns provide an ecological foundation for the observed differences in microbial diversity, biomarker identification, and functional pathway representation.

### Dietary protein source impacts microbial diversity and community structure

Consistent with prior findings [61,62], plant-protein diets were associated with greater microbial alpha diversity, reflecting richer and more evenly distributed microbial communities. The GLMM analysis, which modeled Shannon diversity as the dependent variable, identified dietary protein source as the strongest predictor of alpha diversity, whereas protein concentration (percentage kilocalories), country, sample site, and host age also contributed significantly. The high conditional *R*^*2*^ underscores the combined influence of biological and environmental variables on microbial richness [35,36]. Sequencing depth did not significantly impact diversity estimates, indicating that the observed patterns were not driven by differences in sampling depth [31,32].

Beta diversity analysis revealed further separation in community structure between dietary groups. PCA showed distinct clustering, supported by significant PERMANOVA and ANOSIM results. These findings align with previous studies reporting that plant-based diets support more heterogeneous microbial communities, whereas animal-protein diets tend to favor narrower assemblages enriched in nitrogen-metabolizing taxa [63]. Together, these results indicate that dietary protein source contributes to both microbial richness and the organization of functionally distinct ecological communities [64].

### Microbial biomarkers reflect functional adaptation to protein type

Machine learning analyses identified microbial taxa that reliably distinguished between plant- and animal-protein diets. RF and LEfSe consistently highlighted *Muribaculaceae*, *Faecalibaculum*, and *Parasutterella* as biomarkers of plant-protein conditions, consistent with their known roles in saccharolytic fermentation and nitrogen salvage. *Muribaculaceae* is a dominant and functionally diverse group in murine gut ecosystems, often associated with polysaccharide utilization and propionate production [65]. Its enrichment here supports prior reports linking it to fiber-rich and lower-protein diets. *Faecalibaculum*, another SCFA-producing genus, has been implicated in carbohydrate fermentation and maintenance of gut barrier function [66]. *Parasutterella*, though less frequently studied, has been linked to bile acid metabolism and appears responsive to plant-derived nitrogen sources, suggesting potential roles in amino acid salvage under lower-protein conditions.

In contrast, *Bifidobacterium*, *Streptococcus*, and *Colidextribacter* were consistently enriched in animal-protein diets, reflecting a microbial community adapted to proteolytic substrates. Although *Bifidobacterium* is traditionally associated with fiber metabolism, certain species are capable of amino acid fermentation and expand in protein-rich, low-fiber contexts [67]. *Streptococcus* may utilize simple nitrogenous compounds and peptides from dietary or host sources [68], whereas *Colidextribacter* has recently been associated with branched-chain and aromatic amino acid fermentation in murine models [69].

Although RF provided high predictive accuracy, LEfSe identified taxa with the strongest enrichment and biological effect sizes [38]. Together, these findings suggest that dietary protein type selectively enriches microbial lineages with distinct metabolic strategies. Our study builds on prior research by linking taxonomic signatures to predicted metabolic pathways, highlighting these taxa as potential biomarkers of diet-associated functional shifts in the gut ecosystem [40].

### Dietary protein source shapes gut microbial metabolism and functional networks

Predicted functional profiling revealed that plant- and animal-based protein diets were associated with divergent metabolic tendencies within the gut microbiome. Plant-protein diets were linked to increased representation of pathways involved in carbohydrate metabolism, nitrogen recycling, and aromatic amino acid transformation, including the Entner-Doudoroff pathway, nitrate reduction, and tryptophan degradation. These pathways are commonly associated with metabolic flexibility and efficient substrate utilization in environments with greater carbohydrate availability and relatively lower nitrogen load [70,71].

By contrast, animal-protein diets favored pathways related to proteolytic fermentation, BCAA metabolism, and sulfur-containing compound processing, including creatinine degradation and BCAA biosynthesis. Such metabolic profiles are characteristic of nitrogen-rich environments and have been linked to the production of metabolites such as ammonia, branched-chain fatty acids, H_2_S, and phenolic compounds [6,72]. Functional inference in this study is derived from genus-level patterns and predicted pathway representation based on 16S rRNA profiles, providing a comparative view of metabolic tendencies across dietary conditions rather than direct measurement of microbial activity.

Network analyses further illustrated how dietary protein source organizes microbial functional interactions. Plant-protein–associated networks were centered on taxa such as *Muribaculaceae* and *Desulfovibrio*, which occupied central positions within subnetworks enriched for SCFA and sulfur-related pathways. In contrast, animal-protein networks highlighted taxa such as *Lactobacillus* as key hubs within amino acid and sulfur biosynthesis modules. These network structures suggest that dietary protein source not only alters microbial composition but also reconfigures functional relationships among taxa, shaping the collective metabolic output of the gut ecosystem [73,74].

### Functional implications of protein-driven microbial shifts

Distinct microbial communities shaped by plant- and animal-based protein diets exhibited markedly different metabolic profiles, particularly in pathways involved in SCFA, BCFA, ammonia, sulfur, and phenol metabolism. These differences reflect not only microbial adaptation to the available nutrient substrates but also carry important consequences for host metabolic health and immune regulation.

SCFA metabolism was most strongly enriched in plant-protein-fed microbiomes. Taxa such as *Muribaculaceae*, *Alistipes*, and *Bacteroides*, which were more abundant under plant-protein diets, correlated positively with glutamate degradation and lysine fermentation pathways—key routes for the production of propionate and butyrate. SCFAs are essential for maintaining intestinal barrier integrity, supporting immune regulation, and providing energy to colonocytes [5,75]. Their anti-inflammatory and antitumor properties have been well documented, especially in the context of fiber-rich and polyphenol-containing diets [76,77]. These findings suggest that plant-based proteins potentially promote a more resilient and protective microbial environment.

In contrast, BCFA metabolism was predominantly linked to microbiomes from animals fed animal-based protein diets. Microbes such as *Clostridium sensu stricto* 1, *Colidextribacter*, and *Lactobacillus* were associated with the fermentation of isoleucine and methionine—branched-chain and sulfur-containing amino acids that serve as substrates for proteolytic fermentation. *Clostridium sensu stricto* 1, a genus within the *Clostridia* class, harbors enzymes such as branched-chain aminotransferases and dehydrogenases that enable the conversion of BCAAs into SCFAs like isobutyrate and isovalerate, with ammonia as a byproduct via Stickland fermentation [60,78]. *Colidextribacter*, though recently classified, has been associated in murine studies with the enrichment of genes involved in aromatic and BCAA degradation, suggesting its role in protein catabolism under high-protein diets [79]. Likewise, certain *Lactobacillus* species can catabolize amino acids such as methionine and isoleucine under carbohydrate-limited conditions, utilizing amino acid deamination pathways to generate energy while releasing BCFA and ammonia as end-products [80].

Although BCFAs such as isobutyrate and isovalerate can participate in host signaling, excessive accumulation often coincides with proteolytic fermentation and mucosal stress [81]. These metabolites tend to increase in the absence of carbohydrate substrates, highlighting the trade-off between microbial fermentation strategies and host health outcomes.

Ammonia metabolism followed a similar trend. Animal-protein diets supported the enrichment of Stickland-fermenting taxa, which deaminate amino acids and increase luminal ammonia concentrations. Excessive ammonia is cytotoxic and has been associated with impaired colonocyte metabolism and barrier dysfunction [6,82]. On the other hand, plant-protein diets appeared to favor nitrogen recycling through pathways such as glutamine synthesis. The increased abundance of *Muribaculaceae* and *Parasutterella*, both linked to ammonia assimilation, suggests a microbial adaptation that reduces nitrogen waste and recycles ammonia into biomass [65,83].

Sulfur metabolism further illustrates the contrast between dietary protein sources. Diets rich in animal protein increased the abundance of microbial genes involved in cysteine and methionine breakdown, which are important sources of H_2_S. When produced in excess, H_2_S can interfere with butyrate metabolism, damage cellular DNA, and weaken the intestinal barrier by disrupting tight junctions [84,85]. Microbes such as *Faecalibaculum* and *Lactobacillus*, which played central roles in sulfur-associated networks, likely contributed to elevated H_2_S concentrations in animals consuming animal-based proteins [86]. In contrast, the reduced presence of these pathways in mice fed plant protein may reflect a microbial community that favors alternative fermentation routes, which are less dependent on sulfur metabolism and more supportive of gut barrier function.

Finally, phenol metabolism varied depending on the source of dietary protein. *Colidextribacter*, which was more abundant in mice fed animal-derived protein, showed strong positive correlations with tryptophan and tyrosine degradation pathways (Figure 5B). These pathways are known to produce phenolic metabolites such as *p*-cresol and indoxyl sulfate, which are classified as uremic toxins and have been associated with systemic inflammation, gut barrier dysfunction, and kidney injury [[87], [88], [89]]. These compounds can disrupt gut–liver axis signaling and are increasingly recognized as biomarkers of excessive protein fermentation and microbial imbalance [90]. In contrast, *Bacteroides*, enriched under plant-based protein diets, was correlated with pathways linked to aromatic amino acid metabolism, including those involved in the production of 4-hydroxyphenylacetate (Figure 5B and C). This compound has been reported to exert anti-inflammatory effects and may contribute to metabolic resilience and reduced disease risk [5,91].

Collectively, these findings emphasize that dietary protein type not only shapes microbial taxonomic profiles but also reprograms gut metabolic outputs. Plant-protein diets foster saccharolytic and nitrogen-recycling functions, whereas animal-protein diets enrich for proteolytic and potentially proinflammatory metabolite production. These functional shifts provide mechanistic insights into how dietary protein may influence host physiology, inflammation, and chronic disease risk.

### Implications for diet-microbiome interactions

These findings underscore the ecological and functional adaptability of the gut microbiota in response to dietary protein source. Plant-based proteins were associated with microbial configurations characterized by metabolic flexibility, nitrogen recycling, and pathways linked to saccharolytic fermentation and amino acid utilization. In contrast, animal-based proteins were associated with proteolytic fermentation and biosynthetic routes more commonly linked to nitrogen-rich environments. Notably, animal-protein diets tend to be enriched in sulfur-containing amino acids such as methionine and cysteine, which can be metabolized by taxa including *Clostridium*, *Desulfovibrio*, and *Colidextribacter* into metabolites such as H_2_S and ammonia—compounds that have been associated with mucosal stress and altered gut barrier function [79,92].

Together, these patterns position dietary protein quality as a meaningful axis of microbiome-associated metabolic organization. At the same time, interpretation of protein-specific effects should be considered within the broader dietary context, as co-varying components such as fiber, fat, and phytochemicals differ across dietary patterns and can influence microbial composition and function [64,93,94]. Although this integrative analysis identifies consistent associations between protein source and microbial ecology, variation in overall diet composition likely contributes to the observed signatures. Experimental designs employing matched isocaloric and isonitrogenous diets with standardized nonprotein components will be important for isolating the independent effects of protein source.

Future studies exploring how these microbial shifts intersect with host physiology, including insulin sensitivity, lipid metabolism, immune function, and gut barrier integrity, will further clarify the biological significance of protein-driven microbial shifts, particularly under conditions of metabolic or inflammatory stress. Extending these observations through integrative multi-omics approaches, such as strain-resolved metagenomics, untargeted metabolomics, and host transcriptomics, will enable more direct interrogation of microbial function and provide mechanistic validation of the identified ecological patterns.

### Cross-species relevance and implications for translational nutrition research

Although mice and humans differ in physiology, habitual diet, and baseline microbial composition, several genera were shared across both datasets and displayed broadly similar CLR-based abundance patterns. Genera such as *Brevundimonas*, *Weissella*, *Sphingomonas*, and *Christensenella* appeared consistently across hosts, suggesting that these microbes occupy comparable ecological niches and contribute to similar metabolic functions. These observations align with prior evidence showing that specific fermentative and mucin-associated taxa maintain conserved ecological roles across mammalian gut ecosystems [17,95,96].

Several genera that responded strongly to dietary protein type in mice were also detected at measurable frequencies in human stool samples. Their presence supports the idea that metabolic pathways highlighted in murine protein-intervention models—particularly those related to amino acid fermentation and nitrogen metabolism—reflect ecological strategies not restricted to rodents. This interpretation is consistent with work demonstrating that key functional capacities of gut microbial communities, including amino acid fermentation, are frequently conserved across hosts even when taxonomic profiles differ [18,97].

In this context, murine models offer a controlled and tractable framework for probing nutrient–microbe interactions with relevance for human nutrition. Their genetic uniformity, well-defined diets, and reduced environmental variability allow investigators to isolate the specific effects of dietary components in ways not feasible in free-living human populations. Such mechanistic studies have repeatedly demonstrated value for identifying microbial pathways and phenotypes that are later validated in humans [16,98].

At the same time, the modest ΔCLR differences observed between hosts reflect expected biological variation arising from differences in anatomy, habitual diet, metabolic rate, and lifestyle. These distinctions reinforce that mice should not be viewed as direct surrogates for humans but rather as experimental systems for generating mechanistic hypotheses. The conserved genera identified here provide focused targets for future human studies incorporating strain-resolved metagenomics, metabolomics, and controlled feeding designs to evaluate how protein source shapes microbial metabolism.

### Limitations and recommendations for future research

Although this systematic integrative re-analysis provides insights into how dietary protein sources shape the gut microbiome, several limitations should be acknowledged. First, the use of murine models limits direct translatability to humans, as differences in gut microbial composition, immune responses, and host physiology can influence microbial interactions [17]. Although our cross-species comparison highlights a subset of genera shared between mice and humans, these similarities should be interpreted cautiously, as ecological correspondence does not fully resolve species- or strain-level differences. Nevertheless, murine models remain a cornerstone in microbiome research due to their controlled genetics, defined dietary environments, and tractable experimental design. By focusing on mice, we were able to isolate the effects of dietary protein while minimizing the confounding variables, such as baseline diet variability, medication use, and lifestyle factors, which often complicate human studies. Thus, this analysis provides a foundational framework for identifying microbial and functional patterns that warrant further investigation in human cohorts.

As with most microbiome syntheses relying on publicly available datasets, publication bias cannot be fully excluded, as studies reporting null or negative dietary effects may be underrepresented in the literature. However, because this work reprocessed raw 16S rRNA sequencing data rather than relying on author-reported outcomes or effect sizes, its conclusions are less sensitive to selective reporting of statistically significant results. Accordingly, the findings should be interpreted as identification of consistent ecological patterns rather than quantitative estimation of population-level effect sizes.

Second, this study is inherently constrained by reliance on 16S rRNA gene sequencing and functional inference tools such as PICRUSt2, which limit taxonomic resolution and do not directly measure gene expression or microbial activity. Accordingly, this analysis is explicitly scoped to genus-level ecological structure and inferred metabolic potential derived from 16S rRNA data and does not resolve strain-level functional variation or directly quantify metabolic fluxes. Although such approaches are useful for comparative functional inference across large datasets, the predicted pathways reported here should be interpreted as indicators of metabolic tendency rather than direct evidence of biochemical activity.

Additionally, the absence of fecal or systemic metabolomic data precludes direct validation of microbial metabolic products such as SCFAs, ammonia, H_2_S, and *p*-cresol. As a result, pathway-level inferences should be interpreted cautiously and ideally corroborated using targeted or untargeted metabolite profiling. Although the publicly available datasets included in this study varied in metadata completeness, samples were retained whenever possible, and exclusions were limited to analyses requiring specific metadata fields. All available macronutrient composition data were compiled in Supplementary Table 1 to document variation in diet formulation across studies; however, incomplete reporting limited the incorporation of these variables as covariates in all models.

Future research would benefit from longitudinal human dietary interventions that integrate strain-resolved metagenomics, metatranscriptomics, and untargeted metabolomics to more precisely characterize microbial function and host–microbiota interactions [99]. Evaluating sex-specific and age-dependent responses will further improve understanding of how biological context modulates microbial responses to dietary protein.

Finally, minor methodological differences across studies, such as variation in primer pairs (e.g., 341F compared with 515F), may introduce modest biases in taxonomic profiling. However, the consistent targeting of overlapping hypervariable regions and the use of a standardized bioinformatic pipeline likely mitigate these effects at the level of broader community structure and inferred functional trends. Taken together, despite these limitations, this systematic integrative re-analysis identifies reproducible microbial features and predicted metabolic shifts associated with dietary protein source across diverse murine experimental settings.

In conclusion, this systematic integrative re-analysis identifies dietary protein source as a key determinant of gut microbial diversity, community structure, and inferred metabolic potential in murine models. Across heterogeneous experimental settings, plant-based protein diets were consistently associated with enrichment of saccharolytic and nitrogen-recycling taxa, whereas animal-based protein diets favored proteolytic and sulfur-associated microbial communities, corresponding to distinct patterns in predicted SCFA metabolism, nitrogen turnover, and sulfur-related pathways.

The cross-species analysis indicates that several protein-responsive genera observed in mice are prevalent in human populations, providing contextual insight rather than direct validation of murine dietary effects. Together, these findings highlight dietary protein composition as a modifiable ecological driver of gut microbial organization and functional potential. Rather than establishing population-level effect sizes or direct translational equivalence, this work delineates reproducible microbial patterns that can inform mechanistic hypotheses and guide the design of future controlled, multi-omic human dietary intervention studies focused on protein quality and metabolic health.

## Author contributions

The authors’ responsibilities were as follows – JH-M, SAA: designed the study; SAA, ANO, PD: were involved in data screening and processing; SAA, GL: analyzed the data, created figures, and wrote the manuscript; ANO, JM, CKYL, JMA: reviewed the manuscript; All authors participated in interpreting data, revised the manuscript, and agreed to the published version of the manuscript; and all authors: read and approved the final manuscript.

## Data availability

Data and bioinformatic workflows are publicly available here: https://github.com/deejayprof/META_ANALYSIS.

## Funding

The authors reported no funding received for this study.

## Conflicts of interest

The authors declare no conflicts of interest and affirm that the study’s results are presented transparently, accurately, and free from any fabrication, falsification, or inappropriate data manipulation.
