# Supplementary material for: Dietary Protein Source Shapes Gut Microbial Structure and Predicted Functional Potential: a Systematic Integrative Re-Analysis Using Machine Learning
Source: Adv Nutr. 2026 Jan 2;17(2):100582. doi: 10.1016/j.advnut.2025.100582 (PMC12861163; doi:10.1016/j.advnut.2025.100582)
Supplement: multimedia component 1 [file mmc1.pdf]

Supplementary Materials

Dietary Protein Source Shapes Gut Microbial Structure and Predicted Functional Potential: A Systematic Integrative Re-analysis Using Machine Learning

Samson Adejumo et al.

Supplementary Tables

Supplementary Table 1. 16S rRNA Gene Primers and Regions used in included studies

Country, Year, First Author	Bioproject accession	Target Region	Forward Primer	Reverse Primer
**Australia, 2022 Jian Tan**	PRJEB39583	V3-V4	515F (5′-GTGCCAGCMGCCGCGG-3′)	806R (GGACTACHVGGGTWTCTAAT)
**Japan, 2020 Hiroaki Masuoka**	PRJDB8898	V1- V2	27F (5′-AGAGTTTGATCMTGGCTCAG-3′)	338R (TGCTGCCTCCCGTAGGAGT)
**China, 2022 Yantao Yin**	PRJNA872483	V3-V4	515F (5′-GTGCCAGCMGCCGCGG-3′)	806R (GGACTACHVGGGTWTCTAAT)
**China, 2022 Yifan Zhu**	PRJNA810785	V3-V4	338F (5′-ACTCCTACGGGAGGCAGCAG-3′)	806R (GGACTACHVGGGTWTCTAAT)
**China, 2020 Yunting Xie**	PRJNA607338	V3-V4	515F (5′-GTGCCAGCMGCCGCGG-3′)	806R (GGACTACHVGGGTWTCTAAT)
**Canada, 2021 Béatrice S.-Y. Choi**	PRJEB37442	V3-V4	515F (5′-GTGCCAGCMGCCGCGG-3′)	806R (GGACTACHVGGGTWTCTAAT)
**China, 2020 Muhammad Umair Ijaz**	PRJNA548036	V3-V4	515F (5′-GTGCCAGCMGCCGCGG-3′)	806R (GGACTACHVGGGTWTCTAAT)
**China, 2022 Wuling Zhong**	PRJNA868153	V3-V4	515F (5′-GTGCCAGCMGCCGCGG-3′)	806R (GGACTACHVGGGTWTCTAAT)
**China, 2022 Ranran Zhang**	PRJNA852193	V3-V4	338F (ACTCCTACGGGAGGCAGCAG)	806R (GGACTACHVGGGTWTCTAAT)
**Italy, 2020 Chiara Ruocco**	PRJEB25686	V3-V4	515F (5′-GTGCCAGCMGCCGCGG-3′)	806R (GGACTACHVGGGTWTCTAAT)

Supplementary Table 2. Macronutrient composition (energy-based %kcal) for all rodent diets included in the meta-analysis.

Bioproject accession	Protein (%kcal)	Fat (%kcal)	Carbohydrate (incl. fiber) (%kcal)	Protein type	**Country, Year, First Author**
**PRJEB39583**	60	20	20	Casein	Australia, 2022 Jian Tan
**PRJDB8898**	3	7	81.6	Casein	Japan, 2020 Hiroaki Masuoka
**PRJDB8898**	12	7	71.5	Casein	Japan, 2020 Hiroaki Masuoka
**PRJNA872483**	17.86	2.9	62.95	Oxidized beef	China, 2022 Yantao Yin
**PRJNA810785**	5	4-7	70-80	Wheat starch–based low-protein diet	China, 2022 Yifan Zhu
**PRJNA607338**	20	7.0	62.95	Casein, Pork, Sausage, Soy protein	China, 2020 Yunting Xie
**PRJEB37442**	15	10	75	Casein	Canada, 2021 Béatrice S.-Y. Choi
**PRJNA548036**	20	10.1	69.9	Beef, Casein, Chicken, Pork	China, 2020 Muhammad Umair Ijaz
**PRJNA868153**	61.5	15.9	22.5	Casein	China, 2022 Wuling Zhong
**PRJNA852193**	25	12	63	Branched-chain amino acids	China, 2022 Ranran Zhang
**PRJEB25686**	20	10	70	Essential amino acids, Casein	Italy, 2020 Chiara Ruocco

Supplementary Table 3: Core microbiota based on protein source at the Phylum level

Phyla	Animal Protein (%)	Phyla	Plant Protein (%)
Firmicutes	63.34	*Firmicutes*	47.25
Bacteroidota	19.96	*Bacteroidota*	23.45
Proteobacteria	4.78	*Proteobacteria*	10.21
Actinobacteriota	4.16	*Actinobacteriota*	3.82
Verrucomicrobiota	1.43	*Acidobacteriota*	3.19
Desulfobacterota	1.03	*Chloroflexi*	1.61
Cyanobacteria	0.68	*Cyanobacteria*	1.30
Deferribacterota	0.60	*Verrucomicrobiota*	0.97
Patescibacteria	0.55	*Desulfobacterota*	0.85
Campylobacterota	0.39	*Campylobacterota*	0.66
Others	3.07	Others	6.68

Supplementary Table 4: General Linear Mixed-Effects model comparison

Metric	Model 1	Model 4	Model 5	Model 6	Model 2	Model 7	Model 3
**Number of Parameters (npar)**	4	5	6	7	8	8	9
**Akaike Information Criterion (AIC)**	456.49	417.20	414.68	250.95	356.27	252.88	275.84
**Bayesian Information Criterion (BIC)**	469.15	433.02	433.67	273.11	381.59	278.20	304.32
**Log-Likelihood (logLik)**	-224.25	-203.60	-201.34	-118.48	-170.13	-118.44	-128.92
**Deviance**	448.49	407.20	402.68	236.95	340.27	236.88	257.84
**Chi-squared Test (Chisq)**	-	41.29	4.52	165.73	-	103.39	-
**Degrees of Freedom (Df)**	-	1	1	1	1	0	1
**p-value (Pr(>Chisq))**	-	1.31e-10	0.03356	< 2.2e-16	1	-	1

Models:

M1: Shannon ~ Protein_source + (1 | Isolation_source)
M4: Shannon ~ Protein_source + Protein_level + (1 | Isolation_source)
M5: Shannon ~ Protein_source + Protein_level + Age_in_weeks + (1 | Isolation_source)
M6: Shannon ~ Protein_source + Protein_level + (1 | Country) + (1 | Age_in_weeks) + (1 | Isolation_source)
M2: Shannon ~ Protein_source + Protein_level + Country + (1 | Isolation_source)
M7: Shannon ~ Protein_source * Protein_level + (1 | Country) + (1 | Age_in_weeks) + (1 | Isolation_source)
M3: Shannon ~ Protein_source + Protein_level + Age_in_weeks + Country + (1 | Isolation_source)

Fixed Effects Estimates:

Intercept: 0.33899, Protein_level: 0.40397, Protein_source: 1.25097, Age_in_weeks: 0.30001, Country: -0.55194

Supplementary Table 5: Random Forest Mean Decrease Accuracy Analysis of Top 10 Taxa

Mean Decrease Accuracy	Kingdom	Phylum	Class	Order	Family	Genus
5.344502894	Bacteria	*Bacteroidota*	*Bacilli*	*Lactobacillales*	*Streptococcaceae*	*Lactococcus*
5.094711866	Bacteria	*Firmicutes*	*Bacilli*	*Lactobacillales*	*Streptococcaceae*	*Streptococcus*
4.707552935	Bacteria	*Firmicutes*	*Clostridia*	*Peptococcales*	*Peptococcaceae*	*Peptococcus*
4.432882205	Bacteria	*Firmicutes*	*Bacilli*	*Erysipelotrichales*	*Erysipelotrichaceae*	*Faecalibaculum*
4.437837578	Bacteria	*Actinobacteriota*	*Coriobacteriia*	*Coriobacteriales*	*Atopobiaceae*	*Coriobacteriaceae_UCG-002*
4.349395422	Bacteria	*Firmicutes*	*Bacilli*	*Lactobacillales*	*Streptococcaceae*	*Streptococcus*
4.558971351	Bacteria	*Actinobacteriota*	*Actinobacteria*	*Bifidobacteriales*	*Bifidobacteriaceae*	*Bifidobacterium*
4.465968838	Bacteria	*Proteobacteria*	*Gammaproteobacteria*	*Burkholderiales*	*Sutterellaceae*	*Parasutterella*
4.10452852	Bacteria	*Bacteroidota*	*Bacteroidia*	*Bacteroidales*	*Muribaculaceae*	*Muribaculaceae*
4.060591511	Bacteria	*Bacteroidota*	*Bacteroidia*	*Bacteroidales*	*Muribaculaceae*	*Muribaculaceae*

Supplementary Table 6: Taxonomic Markers Identified by LEfSe for Plant and Animal Diets

Feature	enrich _group	ef_lda	p-value	LDA_Adjusted
Muribaculaceae	Plant	5.233024635	5.40E-06	5.23302463
Allobaculum	Plant	4.64467002	1.88E-09	4.64467002
Bacteroides	Plant	4.565693781	7.05E-08	4.56569378
Escherichia-Shigella	Plant	4.379068769	7.89E-06	4.37906877
Parasutterella	Plant	4.22998311	4.69E-13	4.22998311
Tannerellaceae	Plant	4.188768412	1.77E-10	4.18876841
Lactobacillus	Plant	4.108421192	1.84E-05	4.10842119
Alloprevotella	Plant	4.032406516	3.72E-05	4.03240652
Muribaculum	Plant	3.875563718	1.80E-07	3.87556372
Atopobiaceae_g__	Plant	3.667431414	2.03E-06	3.66743141
Ileibacterium	Plant	3.66050386	2.48E-05	3.66050386
Parabacteroides	Plant	3.658075102	4.33E-08	3.6580751
Tannerellaceae_g__	Plant	3.637155508	3.96E-09	3.63715551
Mycoplasma	Plant	3.512558452	1.24E-13	3.51255845
Lachnospiraceae_UCG-001	Plant	3.486691951	1.06E-12	3.48669195
Helicobacter	Plant	3.430592519	1.74E-08	3.43059252
Erysipelatoclostridium	Plant	3.42541663	0.001001699	3.42541663
Gemella	Plant	3.404947204	1.49E-12	3.4049472
Clostridia_vadinBB60_group	Plant	3.349829721	0.000537424	3.34982972
Cetobacterium	Plant	3.345789548	5.29E-24	3.34578955
UCG-005	Plant	3.322054651	6.64E-13	3.32205465
Peptostreptococcaceae_g__	Plant	3.281207507	1.98E-06	3.28120751
Odoribacter	Plant	3.261282123	0.000978494	3.26128212
Corynebacteriaceae_g__	Plant	3.259661965	5.62E-07	3.25966196
Erysipelotrichaceae_g__	Plant	3.151128193	8.39E-07	3.15112819
A2	Plant	3.147567692	2.25E-05	3.14756769
Chloroplast	Plant	3.074413287	5.77E-20	3.07441329
Neisseriaceae_g__	Plant	3.064649391	1.38E-12	3.06464939

Butyricicoccus	Plant	3.054359327	8.76E-14	3.05435933
Prevotellaceae_UCG-001	Plant	3.047847732	5.63E-16	3.04784773
UCG-010	Plant	3.030970104	5.87E-06	3.0309701
Phascolarctobacterium	Plant	3.020812696	1.46E-17	3.0208127
Pseudomonas	Plant	3.00703619	1.25E-16	3.00703619
Rikenellaceae_g__	Animal	3.292743812	2.42E-07	-3.2927438
Atopostipes	Animal	3.400705568	0.001739201	-3.4007056
Intestinimonas	Animal	3.641121127	0.001865806	-3.6411211
HT002	Animal	3.700185594	0.000483815	-3.7001856
Clostridium_sensu_stricto_1	Animal	3.916651671	4.14E-06	-3.9166517
Colidextribacter	Animal	4.038651745	0.007491941	-4.0386517
Corynebacterium	Animal	4.436842966	0.008381161	-4.436843

Supplementary Figures

Dietary Protein Source Shapes Gut Microbial Structure and Predicted Functional Potential: A Systematic Integrative Re-analysis Using Machine Learning

Samson Adejumo et al.

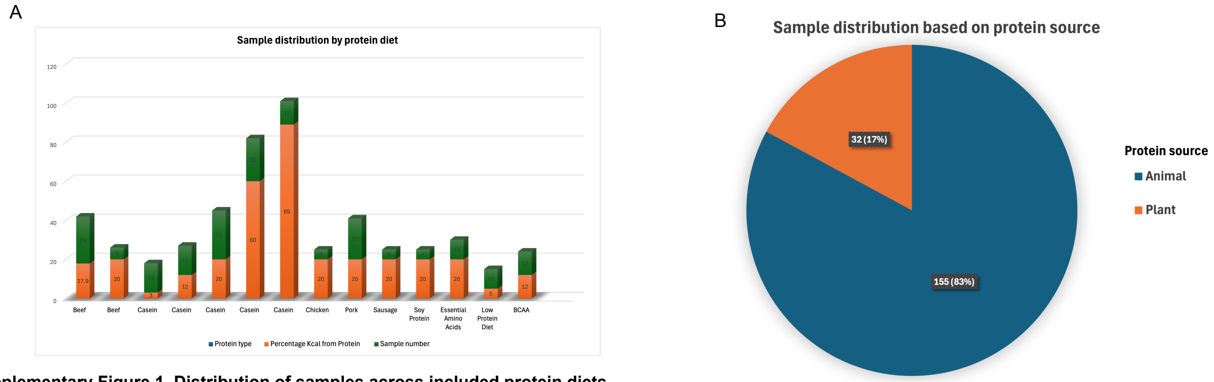

Supplementary Figure 1. Distribution of samples across included protein diets.
(A) Sample distribution by specific protein types, showing protein type, percentage kcal from protein, and total sample number for each diet.
(B) Sample distribution based on protein source (animal vs. plant), illustrating the proportion of samples derived from animal-protein diets (83%) and plant-protein diets (17%).

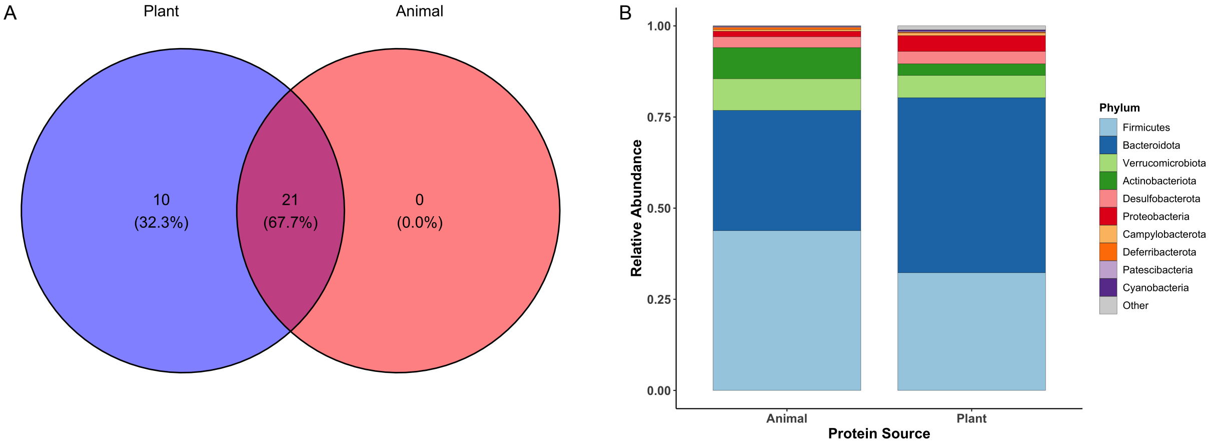

Supplementary Figure 2. Core Microbiota Composition and Unique Microbial Taxa Across Protein Sources at the Phylum Level
(A) Venn diagram showing the number and percentage of shared and unique microbial taxa between plant- and animal-protein diets at phylum level.
(B) Stacked bar plot depicting the relative abundance of dominant phyla across samples grouped by dietary protein source (animal vs. plant).

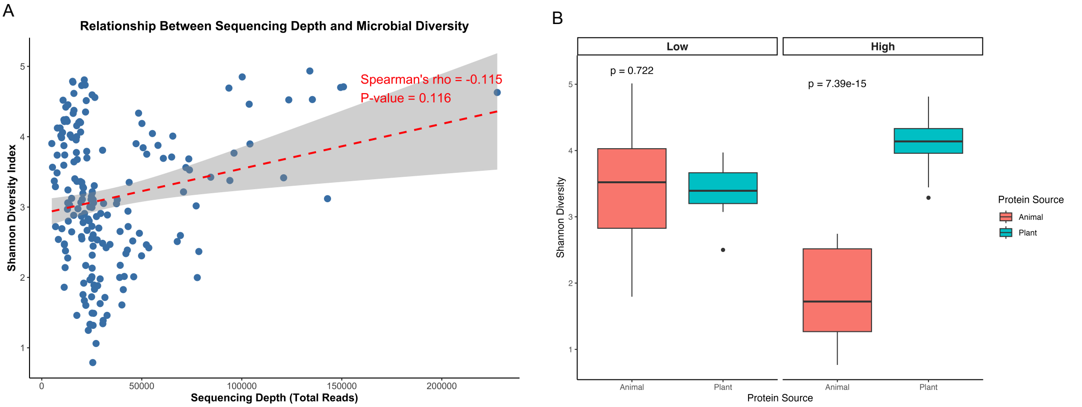

Supplementary Figure 3. Influence of Protein Concentration and Sequencing Depth on Alpha Diversity
A. Relationship between sequencing depth and microbial alpha diversity (Shannon index). Each point represents an individual sample across all studies.
Spearman correlation showed no significant association between sequencing depth and Shannon diversity ($\rho = -0.115$, $p = 0.116$), indicating that observed diversity differences were not driven by sequencing depth.
B. Interaction effect of protein source and concentration on gut microbial diversity (Shannon index). Boxplots show Shannon diversity across low and high protein concentrations for animal- and plant-based diets.
Under high protein conditions, plant-based diets significantly increased microbial diversity compared to animal-based diets ($p = 7.39 \times 10^{15}$). No significant difference was observed at low protein concentrations ($p = 0.722$).

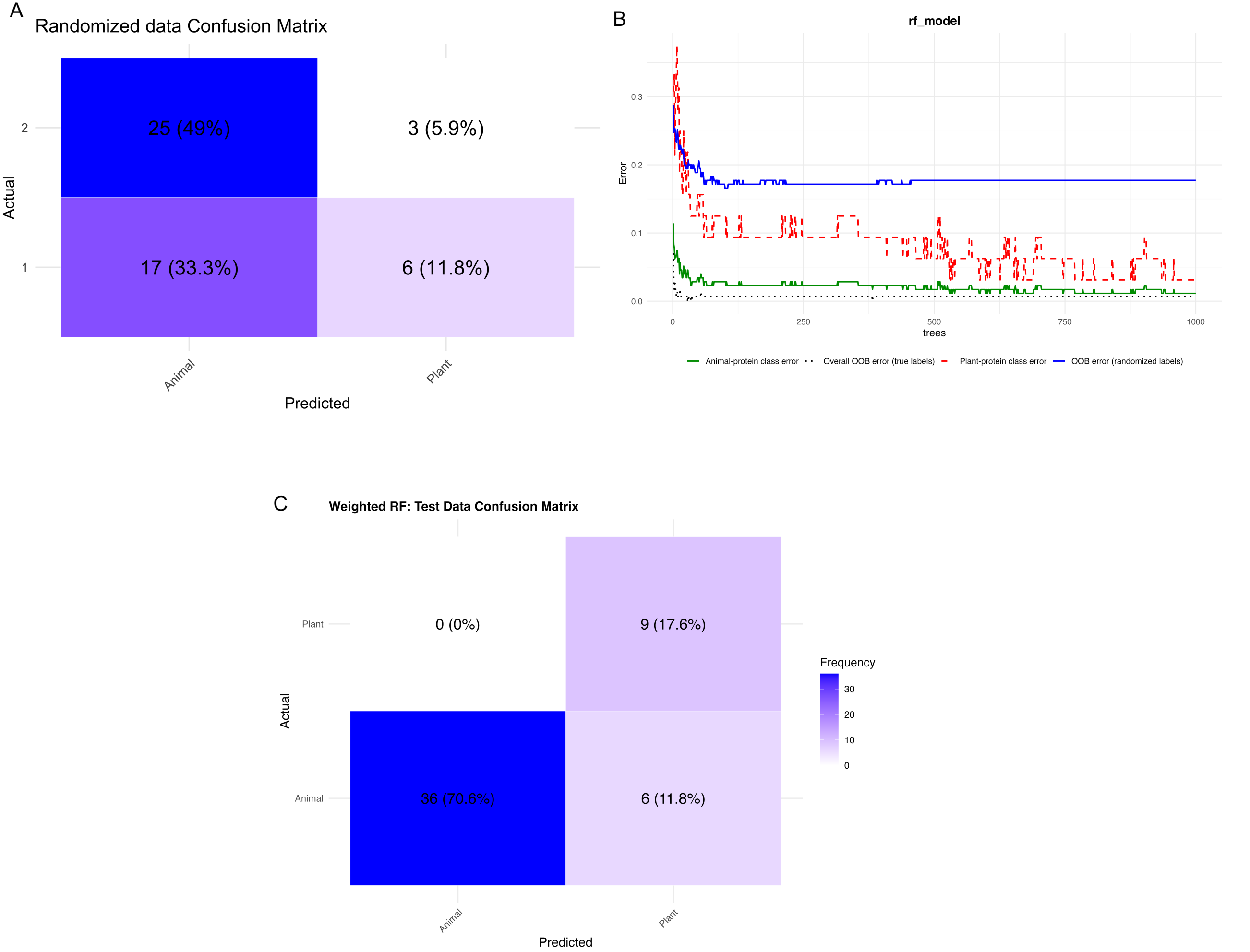

Supplementary Figure 4. Machine Learning Model Randomization and Error Diagnostics
(A) Confusion matrix illustrating the classification performance of the randomized Random Forest model trained on shuffled protein-source labels.
The poor accuracy demonstrates that the original model's performance was not driven by random structure in the data.
(B) Error-rate plot for the Random Forest model displaying classification error across 1,000 trees. The black line indicates the overall out-of-bag (OOB) error, the red dashed line reflects the plant-protein class error, and the green dotted line represents the animal-protein class error.
The stable and low OOB error suggests model convergence and robust predictive performance.
(C) Confusion matrix of the class-weighted Random Forest model showing predictions for the test dataset.
The model achieves high discrimination between plant- and animal-based diets compared with randomized data, confirming true signal.

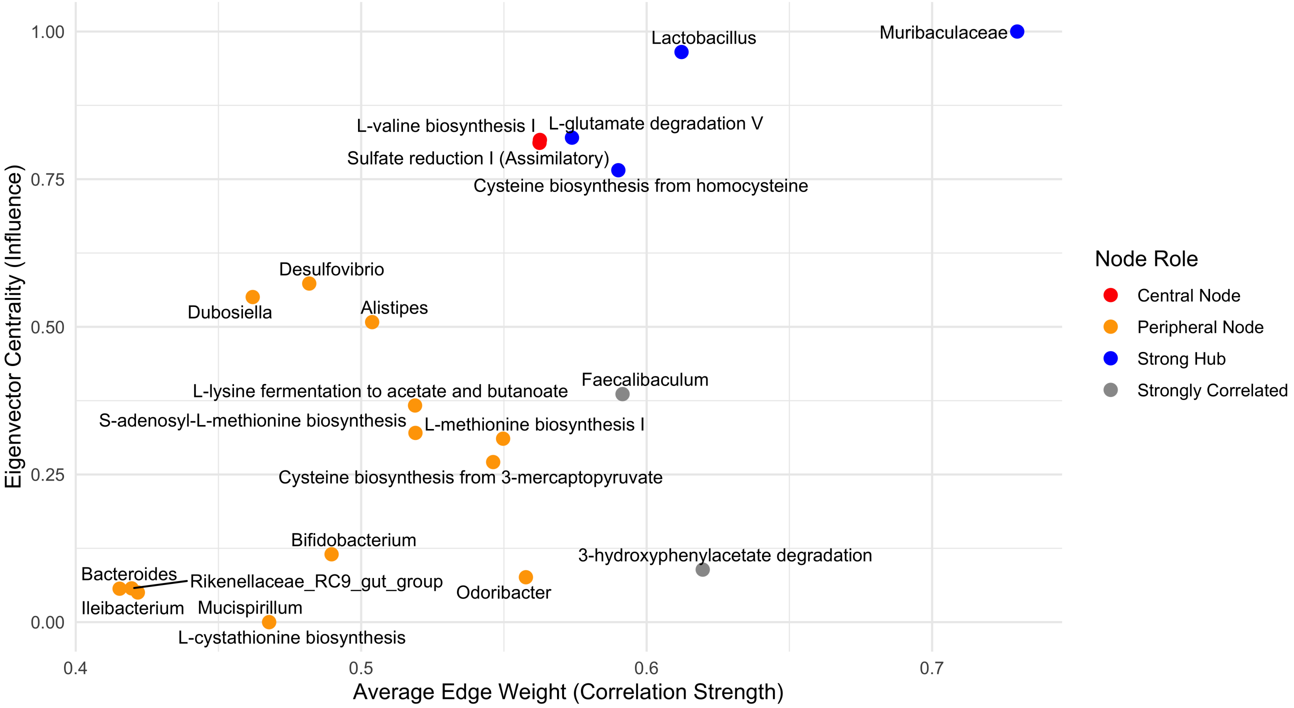

Supplementary Figure 5. Network centrality plot of microbial taxa and predicted functional pathways.
This scatterplot displays node-level topological properties within the taxon–function correlation network.
The x-axis represents average edge weight (correlation strength), and the y-axis indicates eigenvector centrality (node influence).
Node color reflects functional role: blue nodes are strong hubs with high centrality and correlation; red nodes are central connectors; orange nodes are peripheral; and grey nodes are strongly correlated but less influential
